# Photobiomodulation Therapy (PBMT) in Peripheral Nerve Regeneration: A Systematic Review

**DOI:** 10.3390/bioengineering5020044

**Published:** 2018-06-09

**Authors:** Marcelie Priscila de Oliveira Rosso, Daniela Vieira Buchaim, Natália Kawano, Gabriela Furlanette, Karina Torres Pomini, Rogério Leone Buchaim

**Affiliations:** 1 Department of Biological Sciences (Anatomy), Bauru School of Dentistry, University of São Paulo (USP), Alameda Dr. Octávio Pinheiro Brisola 9-75, Vila Nova Cidade Universitária, Bauru, São Paulo CEP 17012-901, Brazil; marcelierosso@usp.br (M.P.d.O.R.); karinatorrespomini@gmail.com (K.T.P.); 2 Medical School, Discipline of Human Morphophysiology, University of Marilia (UNIMAR), Av. Higino Muzi Filho, 1001 Campus Universitário, Jardim Araxa, Marília, São Paulo CEP 17525-902, Brazil; danibuchaim@usp.br (D.V.B.); natalia.kawano@hotmail.com (N.K.); gafurla@hotmail.com (G.F.); 3 Medical School, Discipline of Neuroanatomy, University Center of Adamantina (UNIFAI), Rua Nove de Julho, 730, Centro, Adamantina, São Paulo CEP 17800-000, Brazil

**Keywords:** low-level laser therapy, nerve regeneration, peripheral nerve repair, photobiomodulation therapy, tissue regeneration

## Abstract

Photobiomodulation therapy (PBMT) has been investigated because of its intimate relationship with tissue recovery processes, such as on peripheral nerve damage. Based on the wide range of benefits that the PBMT has shown and its clinical relevance, the aim of this research was to carry out a systematic review of the last 10 years, ascertaining the influence of the PBMT in the regeneration of injured peripheral nerves. The search was performed in the PubMed/MEDLINE database with the combination of the keywords: low-level laser therapy AND nerve regeneration. Initially, 54 articles were obtained, 26 articles of which were chosen for the study according to the inclusion criteria. In the qualitative aspect, it was observed that PBMT was able to accelerate the process of nerve regeneration, presenting an increase in the number of myelinated fibers and a better lamellar organization of myelin sheath, besides improvement of electrophysiological function, immunoreactivity, high functionality rate, decrease of inflammation, pain, and the facilitation of neural regeneration, release of growth factors, increase of vascular network and collagen. It was concluded that PBMT has beneficial effects on the recovery of nerve lesions, especially when related to a faster regeneration and functional improvement, despite the variety of parameters.

## 1. Introduction

Low-level laser therapy (LLLT), now commonly referred to as photobiomodulation therapy (PBMT), using low-level infrared light spectrum lasers is considered a therapeutic advance. Its effects are related to tissue biostimulation, presenting therapeutic responses from photoelectric, photoenergetic, and photochemical reactions [1]. Scientific research has shown the application of PBMT in bone tissue and peripheral nerves with good results whether or not it is associated with other supporting methods in tissue repair [2,3,4,5,6,7].

Laser photobiomodulation presents itself as an electromagnetic technology that is being inserted into clinical practice due to its characteristics that differ from other conventional thermal sources [8,9]. It was observed that there are several features of PBMT that are related to the reduction of tissue repair time and its capacity to increase cell proliferation [10].

In rehabilitative health, PBMT was inserted to promote the repair and recovery of tissues. For example, in physical therapy, the use of PBMT is applied in postoperative phases as an aid in the muscular, nervous, joint, and other functional recovery processes, and in dentistry it is applied in the processes of dental extraction, grafting, osteonecrosis, and periodontal lesions [11,12,13].

The wavelength of infrared irradiation is easily absorbed by tissues and the loss of intensity is minimal, affecting metabolic modifications, DNA activity, adenosine triphosphate (ATP) formation, and the mitochondrial chain. The effect of photobiomodulation is due to the absorption of the photons by cytochrome C oxidase in the mitochondrial respiratory chain, consequently increasing the cytochrome C oxidase activity and therefore ATP formation. ATP from injured or regions of impaired blood perfusion can reactivate injured cells and metabolic disorders [10]. PBMT is also related to pain and inflammation relief and prevention of tissue death to avoid neurological degeneration [14,15].

The wavelength is the key point that regulates the depth and penetration of the laser irradiance in the tissue, noting that the absorption and dispersion coefficients are larger at the lower wavelengths. Regarding the type of wave, whether continuous or pulsed, there are still divergences in which is the best and for which factors are the pulse parameters to be chosen [16]. PBMT presents difficulties in selecting the most suitable parameters for its application due to the lack of standardization, since wavelength, power density, irradiation time, and light polarization have repercussions on the biological effects [9].

Due to the photochemical and photobiological effects of PBMT at the cellular level, there is a relationship between the improvement of trophic conditions and the reduction of inflammatory processes, closely related to a more efficient nervous regeneration and, also, promoting the secretion of neural factors [16,17]. Thus, photobiomodulation therapy in the neurological area acts as an adjuvant in the treatment of traumatic brain degeneration/injury, spinal cord trauma, and in the process of peripheral nerve regeneration.

Peripheral nerve lesions are a reality today, but there is a deficit in relating effective treatments for recovery of the nerves, resulting in considerable functional changes in the daily life of the individual. When injured, the nerve can lose its function, causing motor or sensitive deficits. There is retrograde axonal degeneration to the area of the lesion, so regeneration occurs slowly and sometimes incompletely [18,19].

At the end of the 80’s, the scientific interest in the therapeutic approach of rehabilitation for neural lesions was initiated [20], due to the good results with the use of PBMT in the recovery of injured peripheral nerves but, until the present day, there are still difficulties related to the application parameters [19,20]. Its beneficial effects are independent of the repair technique, neurorrhaphy techniques, and the use of fibrin sealants [3,6,7,21].

PBMT leads to changes in important vascular levels such as elevation of the secretion of antiapoptotic factors in ischemic organs, providing a better wound healing [22,23]; the presence of angiogenesis when ischemic organs were injured [24,25]; a decrease in the site of infarction in rats; as well as elevation in neurological scores following embolic stroke in rats [26,27].

Due to the high range of benefits that PBMT has shown and its clinical relevance of application, the aim of this research was to carry out a systematic review of the scientific papers published in the last 10 years verifying the relation of PBMT with the regeneration of injured peripheral nerves.

## 2. Materials and Methods

A search was performed in the PubMed/MEDLINE database, combining low-level laser therapy AND nerve regeneration keywords, over the last 10 years and restricted to the English language. The next step was to restrict the verification and consultation of articles that used animals as a study object (non-human species).

We verified those articles that presented titles and summaries that approached the subject of this research, as well as methodology, results, and relevance for its practical application.

The articles included should necessarily be presented with full access to the text. The acquired texts were analyzed and synthesized in a reflexive way in order to obtain consistent information on the subject.

## 3. Results

**Figure 1:**
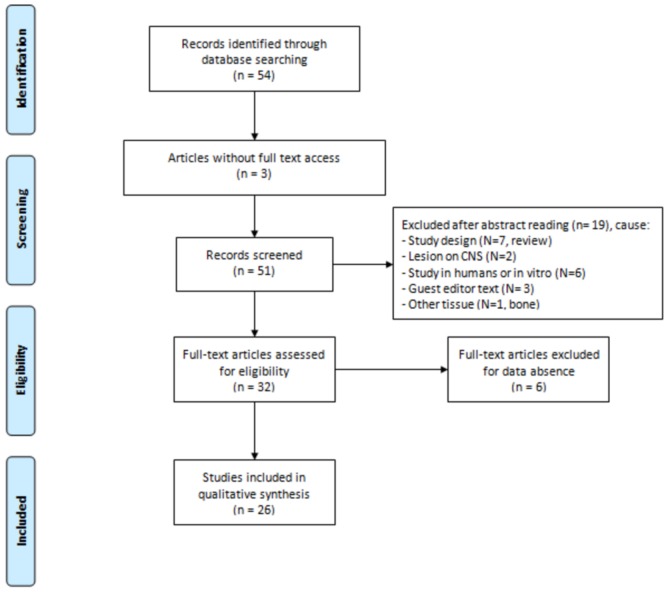
Design used to select the articles.

Initially, 54 articles were obtained from the PubMed/MEDLINE database, of which 28 were excluded because they were not included in the search criteria (in English, study in animals, and full access to content). At the end, 26 articles related to the subject were included. Figure 1 schematizes the search system, according to PRISMA Flow Diagram [28].

Table 1 summarizes the data presented in the 26 articles selected for this research. 

## 4. Discussion

With the evolution of the technology in the health field and the evolution of the adjunct methods for rehabilitation and functional restoration of injured nerves [3,6,7,8,9], the PBMT has shown a wide range of benefits with clinical relevance. Thus, the aim of this research was to carry out a review of the scientific papers published in the last 10 years in order to verify the relation of PBMT in the regeneration of injured peripheral nerves. Regarding the varied benefits of PBMT, the highlight is the reduction of regeneration time and the aid in nerve function.

Among the effects of PBMT on nerve injury, it was verified that the laser minimized the side effects of bupivacaine on the nerve and on the muscle [29], potentiated the process of nerve regeneration observed by morpho-quantitative analysis of the axons and of the nerve fibers [2,3,19,30,31,32,33,34,35], in addition to assisting muscular reinnervation [36].

Photobiomodulation in the nerve injury was also related to a decrease in inflammatory cytokine levels, in pain, and to the facilitation of neural regeneration, demonstrated by the levels of TNF-a, IL-1b, and GAP-43 [32,37].

The functional analysis evidenced the evolution of functional recovery associated with PBMT [6,7,34,38,39]. Marcolino et al. [40] found a functional recovery with both 40 J/cm^2^ and 80 J/cm^2^ (830 nm), Akgul; Gulsoy; Gulcur [41] also scored improvement in functionality with late application PBMT (650 nm) (7 days after injury), as well as Medalha et al. [35] at 660 nm at 50 J/cm^2^. PBMT 660 nm, 10 J/cm^2^, or 60 J/cm^2^ accelerated neuromuscular recovery when compared to 780 nm and 830 nm PBMT [42]. Differently, dos Reis et al. [43] observed that PBMT significantly altered morphometry (myelin sheath thickness values) but did not interfere with the functionality.

Yang et al. [44], when associating PBMT with MSC, demonstrated a better electrophysiological function, immunoreactivity of S100, and fewer inflammatory cells. de Oliveira Martins et al. [45] demonstrated that PBMT (904 nm) had better nociception, greater expression of neural growth factor (NGF) 53% and neurotrophic factor expression (BDNF) 40%. As seen, Gomes; Dalmarco; André [46] evidenced that PBMT (632.8 nm) increased mRNA expression, BDNF and NGF factors after 14 days and maximum expression was observed on day 21. PBMT (660 nm) improved functional index, reduced HIF-1a, TNF-a, and IL-1b, elevated VEGF, NGF, and S100, and decreased tissue ischemia and inflammation [47]. Sene et al. [48] (830 nm) observed that PBMT did not accelerate nerve recovery and the study by Dias et al. [49] when associating PBMT (780 nm) with latex protein also did not find positive results.

The effects of PBMT on nerve damage were verified in the sciatic nerve in 17 articles [19,30,31,32,33,34,35,36,38,39,41,42,43,44,46,47,49], facial nerve in 3 [3,6,7], fibular nerve in 2 articles [40,48], and vagus nerve [2], accessory nerve [29], alveolar nerve [45], and dorsal root [37] in one article each. Of the 26 articles inserted in this review, it was observed that 14 [19,31,32,37,38,39,40,41,42,44,45,46,48,49] presented compression as nerve damage (crushing) and 11 [2,3,6,7,30,33,34,35,36,43,47] articles evaluated the effects of PBMT on neurotmeses, which is the worst type of nerve injury.

It has been observed that the diversity of PBMT application protocols in nerve lesions is large, with the wavelength varying from 632.8 to 904 nm, a varied range of energy and energy density, in addition to the time of application, despite the similarity in the type of lesion targeted in each experiment. As shown, the infrared spectrum has good experimental results. The red spectrum (600 to 700 nm) [50] was seen in 15 studies with satisfactory morphological and electrophysiological results, immunological factors, and tissue markers [2,30,31,33,34,35,36,38,39,41,42,43,44,46,47]. It was also possible to verify the lack of standardization in relation to the application protocols, noting that 6 studies were discarded due to lack of data information regarding energy density and time of application of PBMT.

In a general critical analysis of the articles for the detailed study, a consensus was observed on the effectiveness of PBMT, with the use of low-level laser therapy on the improvement of the morphological and morphometric aspects of the regenerated peripheral nerve, as well as on the reduction of events inflammatory and painful sensitivity, providing faster and higher quality functional recovery [51,52].

In the perspective of new fronts of study, in the last decade, optogenetic and chemogenetic techniques have been used more frequently in the investigation of neuronal circuits, as well as in the study of non-neuronal cells in the brain and peripheral nerves. Optogenetics is effective in generating patterns that mimic neuron responses using a pulse generator that produces lights with different frequencies and pulse durations. Photostimulation can be performed in different subcellular regions, being useful for the study of neuronal circuits in the brain. Chemogenetics are less invasive in animal experiments and do not require the installation of a fiber optic cable into the brain or the connection of the cable to a light source, such as a laser or a light emitting diode (LED).

## 5. Conclusions

At the end of the present study, it can be seen that the data presented in the current articles helped us to understand the beneficial and helpful effects of photobiomodulation on regeneration and functionality after nerve injury. In spite of the great variety of parameters presented, great results were observed, mainly when related to the faster nervous regeneration process.

## Figures and Tables

**Table 1:** Data of selected articles.

Authors	Type of Laser (Manufacturer)	Wavelength (nm)/Spot Beam	Energy (mW)	Energy Density (J/cm^2^)	Radiation Amount	Variables	Irradiation Site	Evaluation Time	Main Results
Buchaim et al. [2]	GaAlAs (Laserpulse IBRAMED, Brazil)	660/0.116	30	4	16 s per point; 3 points	Sural nerve graft was coapted to the vagus nerve using the fibrin glue.	Right side of the neck.	Application on the 1st day post-operatory, 5 weeks, 3 times a week. Evaluation 30 days after irradiation.	LLLT improved the nerve regeneration.
Buchaim et al. [3]	GaAlAs (Laserpulse IBRAMED, Brazil)	830/0.116	30	6	24 s per point; 3 points	Neurotmeses of buccal branch of facial nerve, followed by end-to-end suture or coaptation with heterologous fibrin sealant derived from snake venom	On the surgical site, on both sides of the face	Application 1st day post-operatory, 3 times/week for 5 weeks. Evaluation 5 and 10 weeks after the surgery.	LLLT showed satisfactory results on facial nerve regeneration.
Buchaim et al. [6]	GaAlAs (Laserpulse IBRAMED^®^, Brazil)	830/0.116	30	6.2	24 s per point; 3 points	Neurotmeses of buccal branch of facial nerve, end-to-end anastomosis. Use of epineural suture or coaptation with heterologous fibrin sealant derived from snake venom.	On the surgical site, on both sides of the face	Application on the 1st day post-operatory, 3 times a week, for 5 weeks.	Laser stimulated axonal regeneration accelerated the process of functional recovery of whisker, and the two techniques used allowed the growth of axons.
Rosso et al. [7]	GaAlAs (Laserpulse IBRAMED^®^, Brazil)	830/0.116	30	6.2	24 s per point; 3 points	Neurotmeses in buccal branch of facial nerve, end-lateral anastomosis in the zygomatic branch of the facial nerve with epineural suture or heterologous sealant of fibrin derived from snake venom.	On the surgical site, on both sides of the face	Application on the 1st day post- operatory, 3 times a week, for 10 weeks.	Laser groups presented faster functional recovery, similar results to the control group. It was observed that PBMT provided accelerated morphological and functional repair in the two techniques used.
Ziago et al. [19]	GaAlAs (Twin Laser, MMO, São Carlos, SP, Brazil)	780/0.04	40	4 10 50	4, 10 e 50 s per point; 3 points	Crushing of the left sciatic nerve.	On the surgical site	Application during 6 sessions on alternate days.	Best morphological quantitative and morphometric results on L10 group after 15 days of nerve lesion.
Alessi Pissulin et al. [29]	GaAs (Endophoton, KLD Biosystems, Amparo, Brazil)	904/0.035	50	69	48 s per point	0.5% bupivacaine injection to the right and 0.9% sodium chloride injection to the left on sternocleidomastoid muscle and accessory nerve exposed in surgery.	Ventral side of the neck	Application 1st day post-operatory, during 5 successive days.	LLLT reduced the aggressive effects of bupivacaine on the nerve and the muscle, of muscular degeneration, of myonecrosis and fibrosis, kept the morphology of the axon and the myelin sheath.
Takhtfooladi; Sharifi [30]	GaAlAs (pulsed) LED (red and blue) (-----)	680/0.04 650/1.5 red 450/1.5 blue	10	10	200 s per point; 3 points	Neurotmeses of right sciatic nerve followed by epineural neurorrhaphy.	On the surgical site, sciatic nerve	Application 1st day post-operatory, during 14 successive days	LLLT increased Schwann cells on the great myelinic axons and on neurons, sped up and potentialized nerve regeneration.
Takhtfooladi et al. [31]	InGaAlP (Teralaser; DMC® São Carlos, SP, Brazil)	685/0.028	15	3	10 s per point	Crushing of the left sciatic nerve.	On the surgery site on sciatic nerve.	Application on the 1st day post-operatory, during 21 successive days.	LLLT accelerated and improved the nerve function after crushing lesion.
Wang et al. [32]	GaAlAs (Transverse IND. CO., LTD., Taipei, Taiwan)	808/3.8	170	3 8 15	67.2 s 179 s 335.6 s	Crushing of the right sciatic nerve.	On lesion on sciatic nerve.	Application during 20 successive days.	LLLT (3 and 8 J/cm^2^) accelerated functional and morphologic recovery of the nerve, increased the expression of the marker GAP43.
Shen; Yang; Liu [33]	AlGaInP (Megalas1-AM-800, Konftec Co., Taipei, Taiwan, ROC)	660/-----	0.0032	3.84	5 min per day	Neurotmeses of the left sciatic nerve, 10 mm gap and use of biodegradable tube containing genipin-cross-linked gelatin annexed with β-tricalcium phosphate ceramic particles (genipin-gelatin-tricalcium phosphate, GGT)	Applied to the surgical site.	Application 1st day post-operatory, during 20 successive days. Euthanasia after 8 weeks.	LLLT obtained better functional, electrophysiological and histomorphometric results and assisted on neural repair.
Shen; Yang; Liu [34]	AlGaInP (MegalasVR -AM-800; Konftec, Taipei, Taiwan)	660/----	50	Immediate post-surgery (5.76) 9 following days (0.96)	Immediate post-surgery (30 min) 9 successive consecutive (5 min)	Neurotmeses of the left sciatic nerve, 15 mm gap and the use of 1-ethyl-3-(3-dimethylaminopro-pyl) carbodiimide (EDC) cross-linked gelatin, annexed with β-tricalcium phosphate (TCP) ceramic particles (EDC-Gelatin-TCP, EGT).	On the surgery site.	Application immediately after the lesion, during 9 successive days. Euthanasia after 12 weeks.	LLLT showed better results on the functional index, on development, on electrophysiology, on nerve regeneration, larger neural tissue area, larger axon, and myelin sheath diameter.
Medalha et al. [35]	GaAlAs (Teralaser, DMC São Carlos, São Paulo, Brazil)	660/0.028 808/0.028	30 30	10 e 50 10 e 50	9 s and 47 s; 3 points 9 s and 47 s; 3 points	Neurotmeses of the sciatic nerve, approximately 3 mm distal to the tendon of the internal obturator. Anastomosis with 3 sutures using nylon monofilament 10-0.	Applied to the surgical site.	Application 1st day post-operatory during 5 successive days and 2 days interval until completing 15 days.	LLLT 808 nm on 50 J/cm^2^ obtained higher fiber density. LLLT 660 nm on 50 J/cm^2^ presented larger diameters of axons and of fibers of gait functional recovery.
Shen et al. [36]	GaAlAsP (Aculas-AM-100A, Konftec Co., Taipei, Taiwan)	660/0.1	50	2	2 min per day; 2 points at the same time	A biodegradable nerve conduit containing genipin-cross-linked gelatin was annexed using beta-tricalcium phosphate (TCP) ceramic particles (genipin-gelatin-TCP, GGT) with a 15 mm sciatic nerve transection gap.	On the sciatic nerve.	Application 1st day post-operatory during 10 successive days.	LLLT accelerated the nerve regeneration due to the larger neural tissue, larger diameter and thicker myelin sheath, motor function, electrophysiology and muscular innervation.
Chen et al. [37]	GaAlAs (Transverse IND. CO., LTD., Taipei, Taiwan)	808 ± 5/≤0.5	190	8	207 s	Chronic compression on dorsal root ganglion. A thin L shaped needle (0,6 mm of diameter) was inserted 4 mm in the L4 and L5 intervertebral foramen.	On the dorsal root of L4 and L5.	Application 1st day post-operatory, per 8 successive days. Euthanasia 4 e 8 days.	LLLT decreased the levels of inflammatory cytokines and of pain, facilitating the nerve regeneration, demonstrated by levels of TNF-a, IL-1b e GAP-43.
Belchior et al. [38]	GaAlAs (KLD^®^ Endophoton model)	660/0.63	26.3	4	96.7 s; 3 points	Crushing of the right sciatic nerve.	On the surgical site.	Application 1st day post-operatory, during 20 successive days.	LLLT was positive on the functional index after the 21st day.
Barbosa et al. [39]	GaAlAs (Ibramed^®^ Equipamentos Médicos)	660/0.06 830/0.116	30	10 10	20 s 38.66 s	Crushing of the right sciatic nerve.	On the surgical site.	Application 1st day post-operatory, during 20 successive days.	LLLT 660 nm promoted functional recovery in a faster manner.
Marcolino et al. [40]	AlGaAs (Laser Diode, Ibramed)	830/0.116	30	10 40 80	38.66 s 154.66 s 309.33 s	Crushing of the right fibular nerve.	On the right sciatic nerve.	Application immediately after surgery and during the 21 successive days.	40 J/cm^2^ and 80 J/cm^2^ LLLT influenced the functional recovery of the nerve.
Akgul; Gulsoy; Gulcur [41]	Laser diode (model: DH650-24-3(5), Huanic, China)	650/≈0.14	25	10	57 s on 3 points	Crushing of the sciatic nerve.	On the sciatic nerve.	Early group: Application after surgery, up to the 14th day. Delayed group: Application on the 7th day post-operatory and up to the 21st day.	LLLT accelerated nervous recovery. The group with delayed application showed better functional results.
Gigo-Benato et al. [42]	GaAlAs (TWIN LASER; MM Optics, São Carlos, SP, Brazil)	660/0.04 780/0.04	40 40	10, 60 and 120 10, 60 and 120	0.3 s, 1 min and 2 min 0.3 s, 1 min and 2 min; 2 points	Crushing of the left sciatic nerve.	Applied to the surgical site.	Application 1st day post-operatory, during 10 successive days.	LLLT (660 nm, 10 J/cm^2^ or 60 J/cm^2^) accelerated the neuromuscular recuperation.
dos Reis et al. [43]	AlGaAs (KLD^®^; Endophoton model)	660/0.63	26.3	4	96.7 s per point; 3 points	Neurotmeses and epineural anastomosis on the right sciatic nerve.	On the surgical site.	Application 1st day post-operatory, 20 successive days.	LLLT significantly changed the morphometry (myelin sheath), but did not interfere on functionality.
Yang et al. [44]	GaAlAs (Aculas-Am series, Multi-channel LLLT System, Konftec Corp., Taipei, Taiwan)	660/≈0.2	30	9	60 s per point; 4 points	Use of Mesenchymal stem cells (MSC) on the lesion by crushing of sciatic nerve.	On the sciatic nerve	7 successive days.	LLLT+MSC improved the electrophysiologic function, S100 immunoreactivity, less inflammatory cells and less vacuole formation.
de Oliveira Martins et al. [45]	GaAs (Laserpulse-Laser, Ibramed Brazil) pulsado	904/0.1	70 Wpk	6	18 s on 5 points	Pulsed LLLT. Lesion on alveolar nerve, by a hemostatic Crile clamp.	On the sciatic nerve.	10 sessions every 10 days.	LLLT obtained better nociception, higher expression of neural growth factor (NGF) 53% and of expression of neurotrophic factor (BDNF) 40%.
Gomes; Dalmarco; André [46]	HeNe (----)	632.8/0.1	5	10	20 s on 10 points	Crushing of the right sciatic nerve.	On the sciatic nerve.	1st Application 24 h after surgery; 7, 14 and 21 successive days.	LLLT increased the expression of mRNA and the factors BDNF and NGF after 14 days and maximum expression was observed on the 21st day.
Hsieh et al. [47]	GaAlAs (Aculas-Am series, Multi-channel laser system; Konftec, Taipei, Taiwan)	660/≈0.2	30	9	60 s per point; 4 points	Lesion on the sciatic nerve with 4 ligatures, using chromic suture 4-0.	On the surgery site.	Application 7th post-operatory, during 7 successive days.	LLLT improved functional index, decreased HIF-1a, TNF-a, and IL-1b, increased VEGF, NGF, and S100, reduced tissue ischemia and inflammation, helped the nerve recovery.
Sene et al. [48]	GaAsAl (Physiolux Dual, BIOSET, Rio Claro, Brazil)	830/0.02	30	5 10 20	Maximum time of application was 40 s	Crushing of the right fibular nerve.	Application fibular nerve region.	Application immediately after the lesion, during 21 successive days.	LLLT simulation group obtained a larger nerve transverse area; group 10 J/cm^2^ obtained higher density of the fiber. LLLT did not speed up nerve recovery.
Dias et al. [49]	GaAlAs (Mm Twin Laser Optics, São Carlos, Brazil)	780/0.4	30	15	20 s per point; 3 points	Latex protein (F1) on lesion per crushing of sciatic nerve.	On the surgery site, sciatic nerve.	Application per 6 sessions on alternate days.	LLLT associated to the F1 protein did not present positive results and did not potentialize the effects of this protein.
